# Variation, Variegation and Heritable Gene Repression in *S. cerevisiae*

**DOI:** 10.3389/fgene.2021.630506

**Published:** 2021-03-04

**Authors:** Kholoud Shaban, Safia Mahabub Sauty, Krassimir Yankulov

**Affiliations:** Department of Molecular and Cellular Biology, University of Guelph, Guelph, ON, Canada

**Keywords:** phenotypic heterogeneity, diversity, long non-coding RNA, chromatin, gene regulatory circuits, gene repression, gene silencing

## Abstract

Phenotypic heterogeneity provides growth advantages for a population upon changes of the environment. In *S. cerevisiae*, such heterogeneity has been observed as “on/off” states in the expression of individual genes in individual cells. These variations can persist for a limited or extended number of mitotic divisions. Such traits are known to be mediated by heritable chromatin structures, by the mitotic transmission of transcription factors involved in gene regulatory circuits or by the cytoplasmic partition of prions or other unstructured proteins. The significance of such epigenetic diversity is obvious, however, we have limited insight into the mechanisms that generate it. In this review, we summarize the current knowledge of epigenetically maintained heterogeneity of gene expression and point out similarities and converging points between different mechanisms. We discuss how the sharing of limiting repression or activation factors can contribute to cell-to-cell variations in gene expression and to the coordination between short- and long- term epigenetic strategies. Finally, we discuss the implications of such variations and strategies in adaptation and aging.

## Introduction

Phenotypic variation within a population of single cells can be disadvantageous under benign conditions, but contributes to the optimal fitness of the cell community and projects better survival upon the encounter of an adverse environment (Levy, 2016). At the molecular level, this strategy is implemented by the unnecessary expression of various genes that do not operate under the said benign conditions. Consequently, individual cells “diversify” their investment in gene expression programs. This “bet-hedging” approach is serving to prepare the population in the expectation of a change.

Multiple studies have demonstrated that genetically identical *S. cerevisiae* cells grown under identical conditions display considerable cell-to-cell variations in the expression of individual genes. Importantly, these variations are not produced by mutations in DNA and can be preserved through a significant number of mitotic divisions. Three major mechanisms are known to contribute to such epigenetically transmissible states. The cytoplasmic partition of prions and other unstructured proteins has recently been shown to contribute to quasi-stable heritable traits and to the phenotypic diversification and adaptivity of cell populations. The advances in this direction have been recently reviewed (Harvey et al., 2018) and will not be discussed in detail. This topic will be mentioned only in relation to the role of the *SWI1* prion in modulating gene expression via chromatin structure. Here, we focus on the epigenetic memory of the “on” and “off” states of individual genes as determined by chromatin-mediated gene silencing or by the mitotic transmission of proteins involved in gene regulatory circuits. We scrutinize these phenomena and point out mechanisms that can produce cell-to-cell variations in gene expression and heterogeneity of the cell population. We discuss how a limited abundance of chromatin and/or transcription factors shared by various genes can generate alternating “on” and “off” states of numerous genes. We also examine how the frequency of conversions between “on” and “off” states of genes can change under different conditions or upon aging and how these frequencies can improve the adaptivity of the population.

The material presented in this review is derived from diverse specialized fields of study. These fields often use terminology that describes similar but not necessarily identical phenomena. Here, we use the terms “meta-stability” or “variegation” to refer to the multigenerational epigenetic stability of the “on” or “off” state of individual genes. The stable or meta-stable “off” state of these genes is referred to as “gene silencing.” We use “position-dependent” to describe such epigenetic phenomena when they are dependent on the position of the genes and “position-independent” when they are dependent on the promoters of the genes. The term “bi-modal expression” is used to describe co-existing “on” and “off” states in the same population of cells, however, these on/off states do not display long-term stability.

## Position Effects and Meta-Stability

**FIGURE 1:**
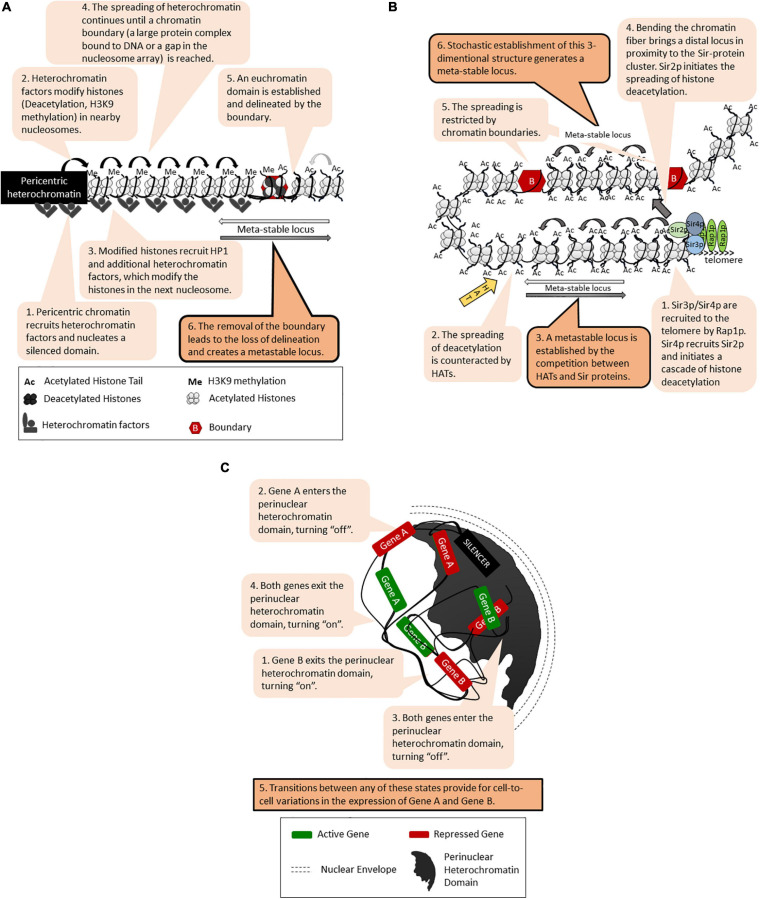
Models for meta-stable gene expression. The figure is available online as an animated PowerPoint^®^ file to better present the sequence of events in the described processes. **(A)** A general model for meta-stability in eukaryotes: Position-Effect Variegation (PEV) at the peri-cenrtic heterochromatin of *Drosophila.* **(B)** Continuous and discontinuous spreading of histone deacetylation, chromatin boundaries and meta-stability at the telomeres of *S. cerevisiae*. **(C)** Epigenetic variations and meta-stability by recruitment into a nuclear heterochromatin domain.

Position effects [called Position Effect Variegation (PEV)] have been initially revealed in *Drosophila* as the patchy red/white appearance of the eye upon the translocation of the *white* gene next to the pericentric heterochromatin (Elgin and Reuter, 2013; Bughio et al., 2019; Figure 1A). Subsequently, many loci in *Drosophila* and other eukaryotes have displayed similar unstable phenotypes that are tightly linked to the position of the locus rather than to the nature of the gene promoters (Elgin and Reuter, 2013; Yankulov, 2013; Sorida and Murakami, 2020). In many cases, the genes within these loci randomly acquire an “on” or “off” state, which is then propagated through multiple generations with infrequent switches between the two states. It is believed that similar transition states and switches drive cell fate decisions during metazoan development. In budding yeast, position effects with similar meta-stability have been observed at the sub-telomeres [called Telomere Position Effect, (TPE)] (Figure 1B) and at the *rRNA* gene arrays (Rusche et al., 2003; Yankulov, 2013; Gartenberg and Smith, 2016). The genes in the mating type *HML* and *HMR* loci are normally completely silenced but display similar on/off phenotypes when silencing is compromised (Rusche et al., 2003). At all these loci, gene silencing is executed by the Sir family of proteins through histone deacetylation and the assembly of tightly packaged chromatin (Figure 1B). The mechanisms of *SIR*-dependent silencing, TPE and PEV have been thoroughly described in several excellent reviews (Rusche et al., 2003; Gartenberg and Smith, 2016; Wang and Elgin, 2019) and will not be detailed here. To date, several models have been introduced to explain the meta-stability of gene expression at these positions (Grunstein, 1997; Fourel et al., 2004; Talbert and Henikoff, 2006; Elgin and Reuter, 2013; Yankulov, 2013; Bughio et al., 2019; Wang and Elgin, 2019). All of them include a *cis*-element for the nucleation and assembly of silencing factors (often called a “silencer”) followed by continuous or discontinuous spreading of the silencing factors (Figures 1A,B). DNA-bound transcription activators and other *cis*-acting anti-silencers or chromatin boundaries can hinder the spreading. These and the competition between *trans-*acting silencing and anti-silencing factors leads to randomly formed “on” or “off” states of the genes at these loci (Figure 1B). Alternative three-dimensional folding of chromatin and the recruitment of the locus into a heterochromatin-rich nuclear subdomain can also contribute to varying on/off states of genes (Figure 1C). It is important that once established, the chromatin structures contributing to the on/off states are propagated via a replication-coupled nucleosome disassembly/reassembly mechanism and the recycling of the existing epigenetic marks on the “old” histones (Almouzni and Cedar, 2016; Stewart-Morgan et al., 2020). However, as discussed later on, the perturbation of histone recycling at the fork can also lead to meta-stability.

## Position-Independent Meta-Stability

Multiple position-independent gene repression mechanisms have been described in *S. cerevisiae* (Sauty et al., 2020). In most cases, it is unclear if gene repression is uniform in all cells of the culture and/or if the repressed states are mitotically transmissible. In addition, many of the repressed genes share similar or the same repressors and co-repressors. It is not clear how these repressors and co-repressors are distributed between the repressed loci and how different genes compete for them. Here, we present examples of known cases of position-independent meta-stable gene expression.

**FIGURE 2:**
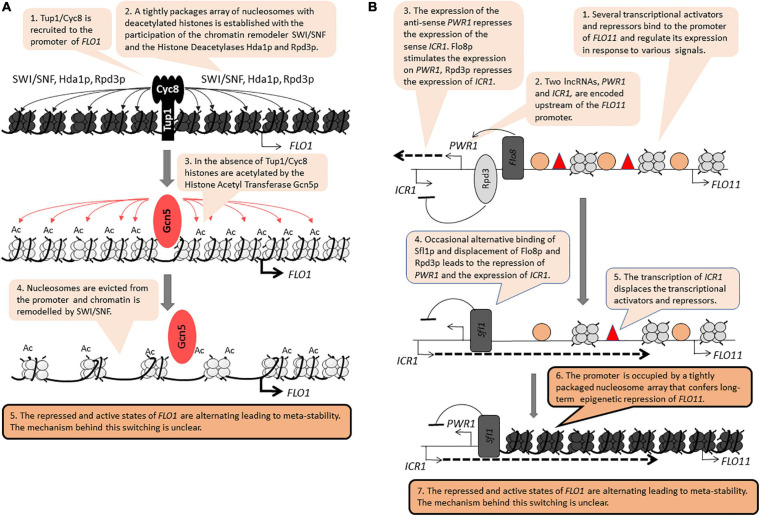
Position-independent meta-stability at *FLO1* and *FLO11*. The figure is available online as an animated PowerPoint^®^ file to better present the sequence of events in the described processes. **(A)** *FLO1*. **(B)** *FLO11*.

The *FLO* genes (*FLO1*, *FLO5*, *FLO8*, *FLO9*, *FLO10*, *FLO11*) encode leptin-like proteins engaged in filamentous growth, cell-to-cell contacts and the formation of biofilm. These genes are positioned 20–40 kb away from the telomeres (Halme et al., 2004; Van Mulders et al., 2009). In laboratory strains derived from *S288C*, the *FLO* genes are stably repressed by tightly packaged chromatin involving the activity of the RSC chromatin remodeler, the Histone Methyltransferase *SET1* and the Histone Deacetylases *HDA1* and *HST1* (Halme et al., 2004; Dietvorst and Brandt, 2010; Fleming et al., 2014). In industrial strains, the *FLO* genes are readily expressed and are regulated by a complex network including the MAPK, TORC, SNF1, and RIM101 signaling cascades (Ryan et al., 2012; Voordeckers et al., 2012). Significantly, the repression of the *FLO* genes is independent of the *SIR* genes (Rowlands et al., 2019). Nevertheless, at least two members of this family, *FLO1* and *FLO11*, are reversibly switching between “on” and “off” states to generate a meta-stable expression pattern, a feature reminiscent of PEV and TPE (Halme et al., 2004; Smukalla et al., 2008; Octavio et al., 2009). At *FLO1*, the Tup1p/Cyc8p co-repressor, the SWI/SNF remodeler and the Histone Deacetylases Hda1p and Rpd3p all participate in the assembly of a tightly packaged nucleosome array over its promoter (Figure 2A; Fleming et al., 2014). This activity is countered by the Histone Acetyl Transferase Gcn5p, which culminates in the eviction of histones from the *FLO1* promoter and the activation of the gene (Figure 2A; Church et al., 2017). These two studies have not revealed how the action of the opposing repression and activation factors lead to the meta-stable state of *FLO1*.

*FLO11* contains a complex promoter and is regulated by multiple transcription activators and repressors (Octavio et al., 2009). Upstream of this promoter, the synthesis of two long non-coding RNAs (lncRNA) contribute to the transition between the on/off states (Figure 2B; Bumgarner et al., 2009, 2012). The synthesis of the anti-sense *PWR1* transcript and the recruitment of the Histone Deacetylase Rpd3p precludes the synthesis of the sense *ICR1* transcript. In contrast, the recruitment of the repressor/activator Sfl1p turns off *PWR1* and allows the expression of *ICR1*. The synthesis of *ICR1* leads to the displacement of transcription factors from the *FLO11* promoter, the assembly of a tight array of nucleosomes and the long-term repression of this gene (Figure 2B). Still, it is not known if the synthesis of the two lncRNAs *per se* contributes to the switching between the “on” and “off” states and if other mechanisms are involved. For example, mutations in genes encoding for histone chaperones that associate with the replication fork lead to meta-stable expression of *FLO11*, but it is not clear how these effects are linked to the synthesis of *PWR1* and *ICR1* (Rowlands et al., 2019).

The conversion rates between the silent and active states of *FLO1* and *FLO11* have not been precisely measured. However, analysis of their activity with GFP-reporters suggest long-term meta-stability similar to the one observed at the sub-telomeres (Smukalla et al., 2008; Rowlands et al., 2019).

The *PHO* genes are controlled by a regulatory network, which is governed by the availability of intracellular phosphate (Korber and Barbaric, 2014). There is a well-documented cell-to-cell heterogeneity in the expression of *PHO84* and of low affinity or high affinity phosphate transporters (Thomas and O’Shea, 2005; Wykoff et al., 2007; Castelnuovo et al., 2013). The expression of *PHO84* is reduced up to 20 fold upon chronological aging (Camblong et al., 2007). Upon transfer to exponential growth *PHO84* maintains its repressed state for at least 24 h, but the expression of the adjacent neighboring genes is not affected (Camblong et al., 2007). Like the repression of *FLO1*, the repression of *PHO84*, *PHO5*, and *PHO8* depends on the recruitment of the Histone Deacetylases Hda1p and Rpd3p and the SWI/SNF remodeler, while their activation involves histone eviction from their promoters (Korber and Barbaric, 2014). Furthermore, the synthesis of an antisense lncRNA over the ORF of *PHO84* has been implicated in the long-term memory of this gene and in the cell-to-cell variations in its expression (Camblong et al., 2007, 2009). Specifically, *PHO84* sense or antisense transcripts are never co-expressed in individual cells, suggesting a switch-like regulation mechanism of expression of these two RNAs (Castelnuovo et al., 2013). The synthesis and accumulation of *PHO84* antisense RNA is paralleled by the recruitment of the Hda1p over the locus, by histone deacetylation at the promoter and by transcriptional repression of the gene (Camblong et al., 2007; Castelnuovo et al., 2013).

The above examples suggest the existence of similar mechanisms that lead to meta-stable variations in the expression of the *FLO* and *PHO* genes. It seems apparent that, in these situations, the synthesis of lncRNAs antagonizes the “on” state. It remains unclear if and how these lncRNA communicate with the factors that modify chromatin and maintain the “off” state. Further analyses of these phenomena are likely to reveal how lncRNAs contribute to rare stochastic conversions and if they participate in the maintenance of the “on” and “off” states.

### Mitotic Transmission of Epigenetic State Through Replication-Coupled Reassembly of Chromatin

**FIGURE 3:**
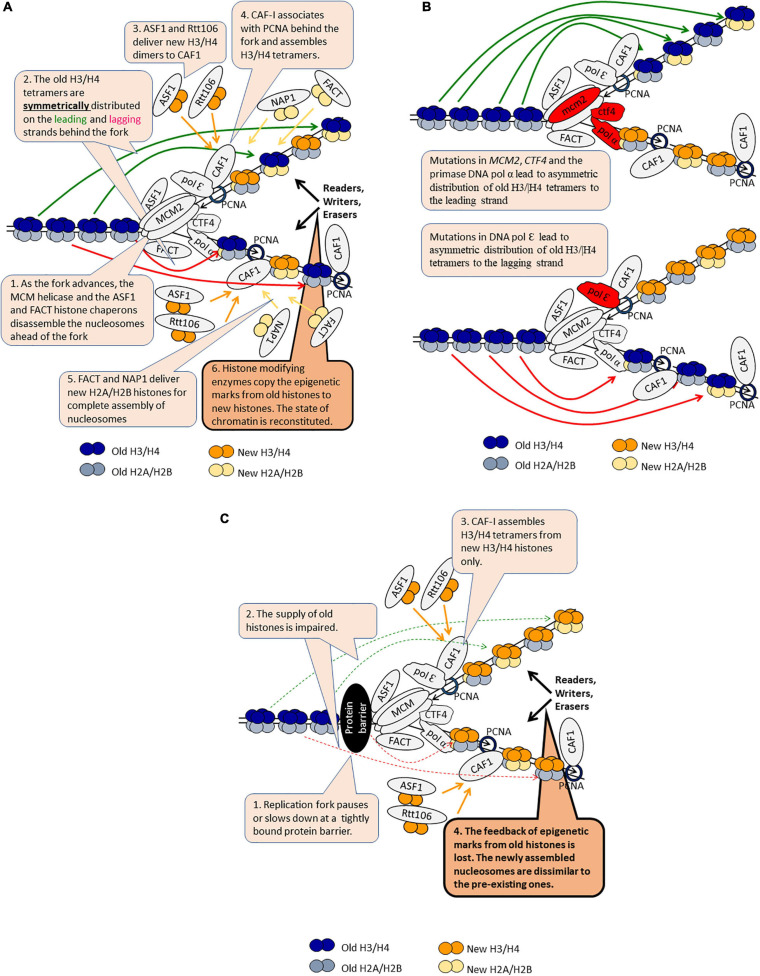
DNA replication-coupled chromatin disassembly and reassembly. The figure is available online as an animated PowerPoint^®^ file to better present the sequence of events in the described processes. **(A)** A model for DNA replication-coupled chromatin disassembly-reassembly and symmetric distribution of old H3/H4 tetramers. **(B)** Asymmetric distribution of old H3/H4 dimers in various mutants. **(C)** Loss of epigenetic marks as the fork pauses or slows down.

As mentioned previously, the epigenetic stability of a locus is governed by the faithful transmission of the established state of chromatin through multiple cell divisions. Eukaryotes have evolved an elaborate replication-coupled system to transmit the existing epigenetic marks of the locus to the loci of the daughter cells (Figure 3A). We have witnessed significant advances in our understanding of this process, but many aspects remain elusive. The disassembly and reassembly of chromatin has been comprehensively reviewed in excellent earlier papers (Almouzni and Cedar, 2016; Rowlands et al., 2017; Stewart-Morgan et al., 2020) and will not be discussed in this section. Here, we focus on the role of histone turnover and the consequences of perturbation of replication fork integrity and/or movement to the transmission of epigenetic states.

The replisome associates with the histone chaperones ASF1, FACT, and CAF1 (Almouzni and Cedar, 2016). In addition, a histone H3/H4 chaperone function has been demonstrated for the replicative MCM helicase (Stewart-Morgan et al., 2020). ASF1 and FACT associate and travel along with the advancing CMG (Cdc45-MCM-GINS) helicase complex (Figure 3A). The joint action of these chaperones and the helicase disassemble the nucleosomes ahead of the fork (Groth et al., 2007; Almouzni and Cedar, 2016). The disassembled H2A/H2B dimers are believed to be transferred behind the fork by FACT, but solid evidence to support this notion is not available (Almouzni and Cedar, 2016). The majority of the disassembled H3/H4 histones are found in the same nucleosome behind the fork suggesting that the whole tetramer is recycled, however, transient splitting of the H3/H4 tetramers can not be ruled out (Figure 3A; Almouzni and Cedar, 2016; Stewart-Morgan et al., 2020). Two sets of proteins are involved in the even distribution of the old H3/H4 tetramers on the leading and lagging strands (Figure 3B). It has been recently demonstrated that mutations in the Dpb3p and Dpb4p subunits of DNA pol ε polymerase cause a biased inheritance of old H3/H4 tetramers to the lagging strand (Yu et al., 2018). Conversely, mutations in *MCM2*, *CTF4* or DNA pol α primase lead to a biased inheritance of old H3/H4 to the leading strand (Gan et al., 2018). Hence, the deposition of H3/H4 tetramers to the leading strand is mediated by DNA pol ε and the deposition of H3/H4 to the lagging strand is mediated by Mcm2p-Ctf4p-DNA pol α (Stewart-Morgan et al., 2020). One would expect that any malfunction of this sorting mechanism would lead to a loss of epigenetic information on one of the strands and, consequently, to epigenetic instability and conversions (Figure 3B). However, a recent study reported that mutations in *DPB3* and *MCM2* only modestly disturb the inheritance of the silent state of the *HMR* locus and suggested that the fidelity of H3/H4 tetramer inheritance could have a limited effect on the transmission of the silenced state in *S. cerevisiae* (Saxton and Rine, 2019). This apparent controversy needs to be resolved by testing if other loci (subtelomeric genes, *FLO* and *PHO* genes) remain epigenetically stable upon mutations of *DPB3* and *MCM2*.

Chromatin behind the fork is re-assembled from equal amounts of new and old histones (Figure 3A). New H2A/H2B histones are delivered by FACT and another chaperone called NAP1. It is not known if old and new H2A/H2B histones are mixed in the same nucleosome. New H3/H4 histones are delivered as dimers by ASF1 and another chaperone, Rtt106. These histones are intercepted by CAF1, which is traveling behind the fork through its association with the PCNA replication clamp. *In vitro* studies have demonstrated that CAF1 can bind a H3/H4 dimer and that two molecules of CAF1 associate with DNA to assemble a H3/H4 tetramer (Figure 3C; Mattiroli et al., 2017; Sauer et al., 2017). Since CAF1 has only one PCNA binding site (Krawitz et al., 2002), it is not clear how the two molecules of CAF1 are recruited to DNA. Importantly, it is unclear if this mechanism operates with new histones only or if it can also work by the tetramer splitting and reassembly of the old H3/H4 histones (Almouzni and Cedar, 2016). For example, it is possible that at *HMR* and other loci this splitting-reassembly mechanism of old H3/H4 takes over the transfer of whole tetramers, thus explaining the epigenetic stability of this locus in *dpb3* and *mcm*2 mutants.

Following the deposition and assembly of histones, the epigenetic marks on the old histones are copied onto the new ones by a largely unknown mechanism (Almouzni and Cedar, 2016; Rowlands et al., 2017). This process culminates in the preservation of the established epigenetic state of the locus in the daughter cells (Figure 3A). Nevertheless, infrequent active→silent and silent→active conversions are readily observed at meta-stable loci (Jeffery et al., 2013; Wyse et al., 2016; Rowlands et al., 2019). The mechanisms of these conversions are poorly understood, but several lines of evidence suggest that fork pausing or replication stress may trigger epigenetic change. Here, we present examples that support this concept. The deletion of *ASF1* or *CAC1* (the gene encoding the largest subunit of CAF1) leads to the reduction of the frequency of epigenetic conversions at the sub-telomeres (Jeffery et al., 2013). The deletion of the *RRM3* helicase, which operates at paused forks due to the encounter of tightly bound proteins to DNA, has a similar effect (Wyse et al., 2016). Even more, combinations of deletions of *RRM3* with other genes lead to variegated expression of *FLO11*, which is not normally expressed in laboratory yeast strains (Rowlands et al., 2019). One possible explanation of these results is that the slowing down or pausing of the fork at loci with tightly bound proteins interferes with the supply of old H3/H4 tetramers (Figure 3C). In turn, an alternative pattern of nucleosome assembly is employed thus leading to loss of the existing epigenetic marks. This alternative pattern could be the CAF1-dependent dimer-to-tetramer mechanism, now operating with new H3/H4 only (Figure 3C). Support for this idea is coming from a study in human cells (Sirbu et al., 2011). It demonstrated that the amounts of PCNA and CAF1 at Hydroxyurea (HU)-stalled forks decrease after adding HU to the medium, but then reach a steady state level of 20 to 30% of the amounts found in the absence of HU. This pattern was likely due to the unloading of PCNA and CAF-1 from the completed Okazaki fragments (Sirbu et al., 2011). Still, PCNA and CAF1 seem to remain associated with at least the leading strands at HU-arrested forks. It remains to be established if the same situation applies to *S. cerevisiae* at forks that are stalled by tightly bound proteins and if at these positions the incorporation of old histones is reduced.

Recently, it was demonstrated that mutations in *ELG1*, a gene responsible for the unloading of PCNA from the lagging strand, increases the rate of silent→active conversions at the *HMR* locus (Janke et al., 2018). This effect was attributed to the retention of PCNA and CAF1 at the lagging strand thus titrating out CAF1 and reducing its reloading at the advancing fork. In this situation, one would expect that in *elg1* mutants the reassembly of chromatin on the leading strand would be affected to a lesser extent. It remains to be elucidated if this deficiency is affected by the DNA pol ε and MCM2-Ctf4-DNA pol α distribution mechanisms.

## Gene Regulatory Circuits and Bimodal Gene Expression

Numerous genes in *S. cerevisiae* are regulated by transcription factors involved in self-regulatory circuits (Lee et al., 2002). Several well-documented phenomena indicate that the mitotic partition of the components of such self-regulatory circuits can lead to memory of past gene activity and of cell-to-cell variations in gene expression (Broach, 2012; Hsu et al., 2012; D’Urso and Brickner, 2017). This form of “on” and “off” states usually lasts for a limited (2–4) number of generations and is often referred to as “bimodal” gene expression. While the abundance of mitotically transmitted transcriptional activators and repressors are central to memory and bimodality, chromatin structure in the vicinity of promoters is also involved. For example, nucleosome-occupied promoters tend to switch between “off” and “on” states to produce bursts of transcription, in turn leading to the higher noise and cell-to-cell variability in gene expression (Sanchez and Golding, 2013). On the other hand, promoters that have low nucleosome density or nucleosome-free regions produce lower variability because of higher burst frequency (Sanchez and Golding, 2013; Sharon et al., 2014). Another study has indicated that stable versus bi-stable “on/off” gene expression is dependent on the spatial distribution of silencing nucleation sites and on whether these sites generate a single or two interacting silencing gradients (Kelemen et al., 2010). Here, we present the *GAL* genes as a paradigm for the bimodal form of gene expression and describe examples of other genes that could employ similar mechanisms.

### Bimodality and Epigenetic Memory of *GAL* Genes

**FIGURE 4:**
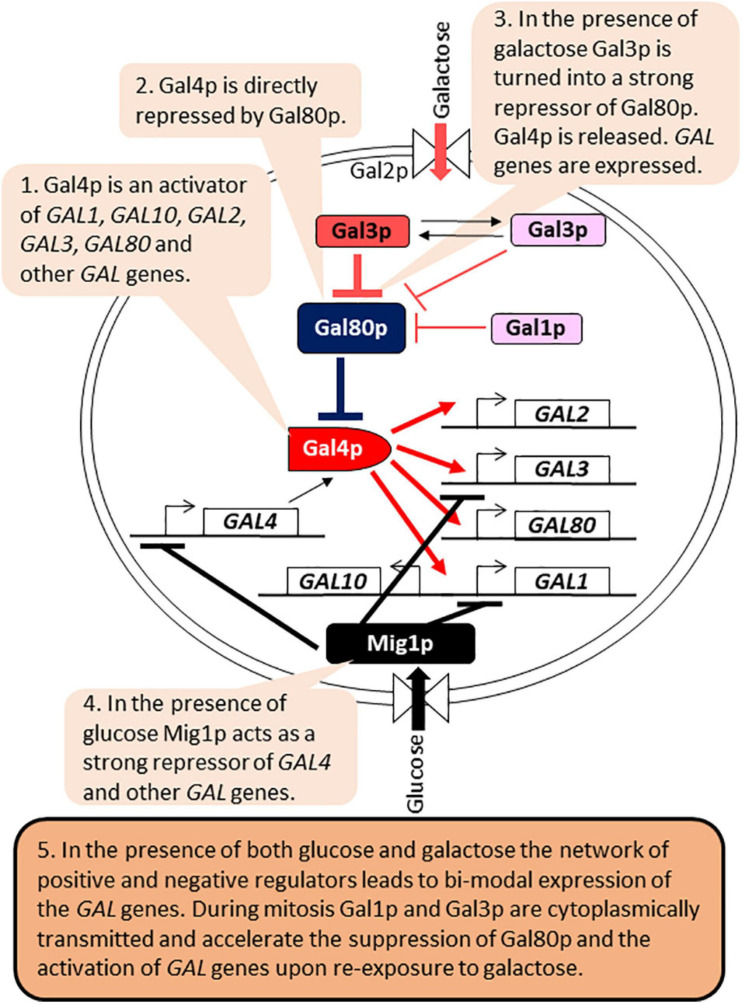
Gene regulatory circuits and bi-modal expression of *GAL* genes. The figure is available online as an animated PowerPoint^®^ file to better present the sequence of events in the described processes.

The control of *GAL* genes is arguably the best-studied example of bimodal expression implemented by a regulatory circuit (Biggar and Crabtree, 2001; Acar et al., 2005; Kundu and Peterson, 2009; Hsu et al., 2012; Venturelli et al., 2015; Figure 4). The regulatory network involves the genes required for the catabolism of galactose (*GAL1*, *GAL2*, *GAL7*, and *GAL10*), which are activated by the Gal4p activator. Gal4p also activates the expression of its own repressor Gal80p, as well as Gal3p, a repressor of Gal80p. In the presence of glucose, Gal4p is inactivated by the Mig1p repressor. In the presence of galactose and absence of glucose, Gal80p is inactivated by Gal3p, leading to the activation of Gal4p and the expression of all *GAL* genes. Gal1p also retains some residual Gal80p inactivation capacity (Venturelli et al., 2015). The regulatory circuit retains an epigenetic “memory” of prior exposure to galactose through the cytoplasmic partition of Gal1p and Gal3p during mitosis. Upon re-exposure to galactose their presence leads to faster inactivation of Gal80p, re-activation of Gal4p and expression of the *GAL* genes (Biggar and Crabtree, 2001; Kundu and Peterson, 2009; Hsu et al., 2012; Figure 4). Long-term memory lasts up to 12 h and is dependent on Gal1p, whose expression is induced more than 1,000 times upon exposure to galactose (Zacharioudakis et al., 2007). Gal3p abundance in galactose increases only 3–5 fold and it has a half-life of 4 h (Kundu and Peterson, 2009). Therefore, Gal3p contributes only to short-term memory, which lasts 1–6 h after the removal of galactose (Kundu and Peterson, 2009). The positive and negative feed-back loops also confer bimodal expression of the *GAL* genes when cells are grown in the presence of limited amounts of galactose. The inherent leakage of the *GAL1-10* promoter, the presence of residual amounts of Gal1p and Gal3p and bursts of stochastic expression of Gal1p all contribute to the random activation of the *GAL1-10* promoter in individual but not all cells in the same culture (Biggar and Crabtree, 2001; Hsu et al., 2012; Venturelli et al., 2012, 2015; Lenstra et al., 2015; Zacharioudakis and Tzamarias, 2017).

While the bimodal expression and memory of the *GAL* genes are heavily influenced by the regulatory circuit, chromatin structure and the synthesis of non-coding RNAs have also been implicated. For example, short-term memory is lost in *swi/snf* mutants (Kundu et al., 2007; Kundu and Peterson, 2009). The most obvious role of SWI/SNF is during re-activation by galactose when the remodeling of chromatin at the *Gal1-10* promoter is a rate-limiting step (Kundu and Peterson, 2010). While *SWI/SNF* seems not to be involved in the memory mechanism *per se*, its activity may also involve the inheritance of distinct chromatin states (Kundu and Peterson, 2010). Consistent with this idea, other studies have demonstrated that the transcription factor Tup1p promotes memory-specific chromatin changes at the *GAL1-10* promoter including the incorporation of histone H2A.Z and the di-methylation of histone H3K4 (Gligoris et al., 2007; Sood et al., 2017). Tup1p functions downstream of Gal1p and seems to promote the loading of a preinitiation form of RNA Polymerase II thus poising the genes for fast reactivation (Gligoris et al., 2007; Sood et al., 2017). Bimodal expression of the *GAL* gene cluster could also involve the synthesis of lncRNA, but the actual effects and mechanisms are somewhat controversial. Earlier studies have shown that the synthesis of an anti-sense lncRNA at 3′ end of *GAL10* recruits Set2p, which in turn methylates H3K36 and recruits the Rpd3L complex to repress the locus (Houseley et al., 2008; Pinskaya et al., 2009). Later studies found that the de-capping of the lncRNAs at the *GAL* gene cluster actually contributes to the rapid and robust induction of the *GAL* gene (Geisler et al., 2012; Cloutier et al., 2013). Two recent studies then demonstrated that the synthesis of the lncRNA is suppressing the inherent leakage of the *GAL1-10* promoter and is inhibiting the transition to the “on” state by repressing the stochastic expression of GAL1 (Lenstra et al., 2015; Zacharioudakis and Tzamarias, 2017).

The *HO* gene represents another case of short-lived “on/off” gene expression where a lncRNA plays a role (Kirchmaier, 2013). *HO* encodes an endonuclease, which initiates the mating type conversion at the *MAT* locus. Next, *MAT* is repaired using the DNA of the silent *HML* or *HMR* mating type loci to produce a switch of the mating type at a frequency of 70% per generation (Kirchmaier, 2013). The *HO* gene is expressed in a brief period in G1 phase in mother cells only. The restriction is enforced by the local translation of Ash1p (a component of the Rpd3L Histone Deacetylase) in daughter cells, which in turn shuts off the *HO* promoter (Pan and Heitman, 2000).

By yeast standards, *HO* has a fairly large promoter spanning 1,900 bases upstream of the initiation site. It is regulated by the Tup1p/Cyc8p co-repressor, the SBF (Swi4p/Swi6p) and Swi5p activators, Rpd3L and the histone chaperones ASF1 and FACT (Parnell and Stillman, 2019). Single-cell analysis of the *HO* promoter has shown a stochastic activation in 2% of the daughter cells and repression in 2% of the mother cells. The “on” and “off” states exhibit short-term (1–2 divisions) of epigenetic memory (Zhang et al., 2013; Yu et al., 2016). The “on” state is memorized by the promoter-bound SBF and Mediator transcription factors, while the “off” state is executed and propagated in *cis* by the synthesis of a lncRNA, which removes these factors from the promoter and renders it inactive (Yu et al., 2016). The authors proposed that nucleosomes and SBF compete for binding to the promoter, resulting in an “on/off” bimodal expression. Mutations in *SWI5*, *ASH1*, and *RPD3* alter the “on/off” rates without affecting the magnitude of *HO* expression, while mutations in other genes affect both the “on/off” rates and the magnitude of *HO* expression (Zhang et al., 2013). Hence, histone deacetylation by Rpd3p and nucleosome repositioning contribute to the rates of on/off transitions of this gene, but details on the mechanism are not yet available.

In summary, it seems that the short-lived on/off states of *GAL* and *HO* genes are influenced by chromatin structure and lncRNA. Some of the involved chromatin factors are also participating in the control of meta-stability at the *FLO* and *PHO* genes. In this regard, it will be interesting to identify factors and mutations that prolong the stability of “on” or “off” states and to analyze what mechanisms link meta-stable epigenetic memory and bimodal short-term variations.

### Memory of Maltose and Inositol Metabolic Genes

Prior exposure to maltose leads to rapid expression of the maltose regulatory genes (New et al., 2014; Cerulus et al., 2018). However, in this situation the transcription memory is not related to a regulatory circuit, but to a decline in the ability of the cells to activate respiration, which in turn is necessary for the utilization of other carbon sources. Similarly, prior inositol starvation induces a transcriptional memory of *INO1*, which encodes inositol-1-phosphate synthase. This effect is associated with the translocation of the gene to the nuclear pore complex and the persistent binding of the Sfl1p activator, as well as with the di-methylation of H3K4 (Brickner et al., 2007; D’Urso et al., 2016). The H3K4me2 histone mark is inherited through several cell divisions and is required for transcriptional memory (D’Urso et al., 2016). It remains to be shown if these two cases of transcriptional memory is accompanied by bimodal expression or if other mechanisms of memory are involved.

### Bimodal Expression of Other Stress-Response, Metabolic and Cell-Cycle Regulated Genes

Metabolic genes in budding yeasts are regulated by a network of signal transduction pathways, which sense the availability of energy and nutrients. The signal transduction pathways often converge on a limited number of shared transcription factors (Petrenko et al., 2013; Gonzalez and Hall, 2017; Gonzalez et al., 2020). Interestingly, nutrient depletion as well as other stresses elicit a common response in a large set of genes, a phenomenon referred to “environmental stress response” (Gasch et al., 2000). Given the bimodality of *GAL* genes, the overlap of signal transduction pathways and the observed common response to environmental stress, one would expect that other metabolic or stress response genes would display similar bimodal variations in gene expression. For example, osmotic stress transiently activates the Hog1p MAP-Kinase, which in turn translocates to the nucleus and activates various genes (Pelet et al., 2011). Interestingly, as Hog1p activity is linearly increasing with the stimulus, multiple Hog1p-responsive genes display bimodal expression through a stochastic process of chromatin remodeling and slow transition from repressed to active state (Pelet et al., 2011). It has also been shown that Casein Kinase II contributes to the stochastic variations in response to osmotic stress (Burns and Wente, 2014). This kinase stimulates the Hot1p transcription activator and at the same time negatively regulates Hot1-responsive genes. Ultimately, bi-modal expression of osmotic stress-responsive genes is abolished upon destruction of Casein Kinase II (Burns and Wente, 2014).

Recent single-cell RNA sequencing experiments support the notion of bimodality stress-responsive genes (Gasch et al., 2017; Nadal-Ribelles et al., 2019). The first study documented gene expression variations in individual cells both before and after exposure to environmental stress (Gasch et al., 2017). The other study demonstrated bimodal gene expression in numerous genes, with cell-cycle and metabolically regulated genes prominently represented in the variably expressed ORFs (Nadal-Ribelles et al., 2019). The heterogenic expression of the metabolic genes was cell-cycle independent, indicating that these two classes of genes do not share common regulators. These studies did not track the length of epigenetic “memory” and the conversion frequency between the “on” and “off” states of the bimodally expressed genes. However, another study suggests that the conversion frequency at the cell cycle regulated genes could be high. It was demonstrated in isogenic cultures that the division times of individual cells significantly vary and that these division times persist through a short (1–2) number of generations (Cerulus et al., 2016). The mechanism of these variations and their epigenetic inheritance is largely unknown. An earlier publication sheds some light on the role of nucleosome occupancy at the promoter of one such gene, *CLN2* (Bai et al., 2011). The *CLN2* promoter binds several sequence-specific factors (Reb1p, Mcm1p, and Rsc3p) which contribute to the formation of a Nucleosome-Depleted Region (NDR). While *CLN2* is robustly expressed in all cells in each cell cycle, the removal of the binding sites for these factors and the loss of NDR indices unreliable “on/off” expression in individual cells (Bai et al., 2011). It seems that the capacity of the cells to form and maintain these NDRs could be a contributing factor to the bimodal expression of *CLN2* and possibly other cell-cycle regulated genes.

In summary, the above studies suggest that stress response, cell-cycle and metabolically regulated genes can alternate between short-lived “on” and “off” states. The interplay between signal transduction pathways, shared transcription factors and regulatory circuits could be the major determinant of the observed cell-to-cell variations of the expression of these genes. At present, it is not known to what extent chromatin regulatory factors are part of this network and if they contribute to the described bimodality and epigenetic memory. Furthermore, it has not been determined if the wide-spread heterogeneity of gene expression shares mechanistic similarities to position-dependent and position-independent meta-stability.

### Transcriptional Repression Memory (TREM)

So far, we have described how cells keep a memory of a past “on” state of genes, and how this memory can lead to bimodal gene expression. A recent study has indicated that cells can also keep memory of a past “off” state (Lee et al., 2018). The authors analyzed the rates of gene activation and repression upon shifting the carbon source from raffinose to galactose to glucose and then back to galactose. Many genes that were not directly involved in galactose metabolism were faster activated during the second exposure to galactose, thus showing memory of their prior active states during the first galactose incubation. At the same time, more than 500 genes were repressed during both rounds of exposure to galactose (Lee et al., 2018). Remarkably, the analysis of selected repressed genes (*REI1*, *TEA1*, and *RRN11)* showed a much faster and stronger repression during the second galactose exposure. These findings indicate that genes can maintain a memory of their inactive states. It was shown that in *rpd3*Δ or *pho23*Δ (another subunit of the Rpd3L complex) mutants, the steady state levels of TREM-affected genes do not change, but their repression upon the second shift to galactose is weaker and slower (Lee et al., 2018). It turned out that Pho23p directly interacts with tri-methylated H3K4 and that the H3K4Me3 mark is necessary for TREM. The authors proposed that H3K4Me3 marks, which are left on the promoters during the intermediate step when TREM genes are active, recruits Rpd3L via the Pho23p subunit. In turn, Rpd3L deacetylates histones, interferes with the recruitment of RNApol II and quickly shuts them off. However, it remains unclear how the memory of the initial repressed state is maintained until the re-exposure to galactose and how H3K4Me3 is used as both an activation and a repression histone mark. Very importantly, an overwhelming number of the genes that display transcriptional repression memory upon repetitive shift to galactose are not regulated by *GAL4* and the *GAL* regulatory circuit. Consequently, the transcriptional memory for most of these genes is maintained by a distinct mechanism.

## Other Examples of Cell-To-Cell Variations in Gene Expression

*GSY1* and *GSY2* encode Glycogen Synthase. Both genes are positioned away from the telomeres and are expressed upon glucose or nitrogen starvation or environmental stress (Hardy et al., 1994; Unnikrishnan et al., 2003; Enjalbert et al., 2004). Glycogen synthesis is normally initiated by glycogenin, which is encoded by *GLG1*/*GLG2*. Interestingly, *glg1Δglg2Δ* strains produced 1–3% of colonies that bypass the glycogenin-dependent step (Torija et al., 2005). The acquired phenotype was stable so that cells from the glycogen^(+)^ colonies produced more than 95% glycogen^(+)^ colonies. Remarkably, staining of these colonies for glycogen produced a segmented pattern with an appearance similar to that observed in cells harboring *ADE2* at the telomere (Torija et al., 2005). These results suggest that *GYS1* and *GYS2* have an epigenetic meta-stable pattern of expression similar to that observed at the telomeres.

A recent study used a model system for fitness-directed stochastic tuning of gene expression (Freddolino et al., 2018). The authors used a weak promoter to drive the expression of a Ura3p-mRuby reporter and observed “tuned” ura + colonies at about 10^–3^ frequency. These “on” states persisted for multiple generations after the removal of the selective pressure. Of note, mutations in *GCN5* (a Histone Acetyl Transferase) or treatment of the cells with Nicotinamide (an inhibitor of Histone Deacetylases) accelerated the reversal to the un-tuned “off” state. The deletion of the histone chaperone *ASF1* slowed down both the acquisition of the tuned state and the reversal to naïve state. All these observations point to a role of chromatin in the maintenance of the acquired “tuned” state.

## Discussion and Hypotheses

### Limited Abundance of Repression and Activation Factors Can Explain Heterogeneity of Gene Expression

Heterogeneity of gene expression in *S. cerevisiae* is produced by “on/off” states of individual genes in individual cells. These states last for 1–2 cell divisions or persist for significantly higher number of generations. These traits are not produced by mutations in DNA but by alternations in chromatin structure or by the mitotic transmission of factors that participate in gene regulatory circuits. In both cases, transcription-permissive or transcription-repressive chromatin structures are involved. In this regard, it is significant that many genes with “on/off” expression states are regulated by diverse mechanisms but share the same limited set of repressive or activation co-factors. The SWI/SNF remodeler represents one such striking example. It is involved in both repression or activation steps and has been detected at the promoters of 5% of the yeast genes (Yen et al., 2012). Upon stress, SWI/SNF association increases at the promoters of 529 genes participating in carbohydrate metabolism, stress response and amino acid synthesis (Dutta et al., 2014). As discussed, many of these genes do or are likely to display bimodal expression. As mentioned earlier, mutations in SWI/SNF also affect the expression of *FLO* genes and gene silencing at the telomeres. On the other hand, the abundance of the SWI/SNF complex was estimated at 100–200 copies per cell (Cairns et al., 1996), promptly raising the question of the genome-wide distribution of SWI/SNF in individual cells and its possible contribution to the heterogeneity in the expression of both meta-stable and bimodally expressed genes. Even more, the Swi1p subunit of SWI/SNF is a well characterized prion (Du et al., 2008; Goncharoff et al., 2018). Its prion SWI^+^ state arises at a frequency of 10^–3^–10^–5^ and leads to the long-term suppression of *FLO1* and *FLO11* and to the concomitant suppression of cells adhesion and pseudohyphal growth (Du et al., 2015). Interestingly, the epigenetic prion switch (SWI^+^) establishes a population of migrating “pioneer” cells while the SWI^–^ cells retain a flocculation-competent “settler” phenotype (Newby and Lindquist, 2017). These observations reiterate the significance of phenotypic heterogeneity for the optimal fitness of the cell population. Importantly, they show a connection between the three mechanisms known to contribute to the heterogeneity and its transmission by epigenetic means.

The Histone Deacetylases *RPD3, HDA1, HST1*, and the Tup1p-Cyc8p complex are other examples of shared co-factors. Some of them have also been implicated in the positional *SIR*-dependent silencing at the telomeres and in the repression of *FLO* and *PHO* genes (Rowlands et al., 2019). The Histone Methyl Transferases *SET1* and *SET2* are positive regulators of 80% of the yeast genes, however, multiple genes are overexpressed in *set1*Δ or *set2*Δ cells (Jaiswal et al., 2017; Woo et al., 2017). The repressive function of *SET1* and *SET2* has been linked to the expression of lncRNAs or anti-sense RNAs and/or to the incorporation of H3K4 and H3K36 methylation marks that are subsequently recognized by co-repressors (Jaiswal et al., 2017; Woo et al., 2017; Lee et al., 2018). Apart from histone modifying enzymes and remodelers, other shared proteins could contribute to stochastic variations of gene expression. For example, large numbers of genes respond to the same external stimuli and therefore share the signal transduction factors (Gasch et al., 2000; Petrenko et al., 2013; Gonzalez and Hall, 2017; Gonzalez et al., 2020).

The impact of the limited abundance of a shared silencing factor to position-dependent variegation has been well demonstrated at the *SIR*-dependent loci. Earlier studies have shown that the enhancement of silencing at the mating type and *rRNA* loci leads to a reduced silencing at the telomeres (Roy and Runge, 2000), while reducing the silencing at the telomeres induced *SIR*-dependent silencing and variegation at an engineered locus away from the telomere (Marcand et al., 1996). Furthermore, elevated expression of SIR proteins induced the meta-stable repression of a telomere-distal *SIR*-dependent reporter that was not normally silenced at physiological levels of the SIR proteins (Marcand et al., 1996). Ultimately, it is the limiting abundance of Sir3p that determines the span and stability of the silenced loci and the variegated pattern of gene expression at the telomeres (Wiley and Zakian, 1995; Grunstein, 1997; Motwani et al., 2012). Similarly, the abundance of Sir2p was shown to be a limiting factor for the silencing of the *rRNA* genes and that telomeres and *rRNA* genes compete for this factor (Smith et al., 1998).

It is conceivable that the sharing of other positive and negative regulators can lead to stochastic variations in the expression of other genes. A vast number of genes including some of the variably expressed genes described earlier, are de-repressed upon deletions of *RPD3, HDA1, HST1, SET1*, or *SET2*, strongly suggesting that these factors are shared by them. If the sharing of co-repressors and co-activators is contributing to the stochastic variations in gene expression, then the overexpression of these genes should suppress the bimodal expression and/or increase the proportion of repressed meta-stable genes. Apart from addressing this fundamental question, such experiments can identify the limiting factors (like Sir3p for the *SIR*-dependent silencing mechanisms) that control bimodality and can provide paradigms for manipulating the adaptivity of cells.

### Regulatory Circuits Versus Transmission of Chromatin Structure

Most of the genes that display bimodal expression have fairly complex promoters. They bind multiple transcriptional activators and repressors and respond to various signal transduction pathways (Broach, 2012; Gonzalez and Hall, 2017). Complex promoters also regulate some of the genes that display position-independent variegation (Broach, 2012). It is given that the interplay between positive and negative signals would provide finely tuned control that culminates in bimodal expression. However, the epigenetic memory of these genes is not completely understood. The in-depth analysis of the *GAL* genes has provided a solid backing to the idea that their transcriptional memory is determined by the regulatory circuit (Hsu et al., 2012). Nevertheless, the influence of histone modifiers and chromatin remodelers on short-term transcriptional memory or bimodality has also been documented (Kundu and Peterson, 2009; Kirchmaier, 2013). It is possible that the faithful transmission of chromatin could be an integral component of both short-term and long-term epigenetic memory. We have recently shown that the repression of *FLO* genes in laboratory strains is relieved by mutations in the *ASF1* and *CAC1* genes (Rowlands et al., 2019). These genes encode histone chaperones, which act in replication-coupled chromatin reassembly (Rowlands et al., 2017). Others have shown that mutations in *CAC1* and *POL30*/PCNA (the replicative clamp) reduce the stability of the robustly silenced mating type loci (Janke et al., 2018). In both cases, the epigenetic effects have been linked to dysregulated transmission of the chromatin state at these loci. It will be interesting to test if genes with bimodal expression that are controlled by regulatory circuits lose bimodality in *asf1*, *cac1*, and *pol30* mutants. Such experiments would address to what extent short-term transcription memory is determined by chromatin structure.

### Frequency of On/Off Conversions and Bet-Hedging Strategies

**FIGURE 5:**
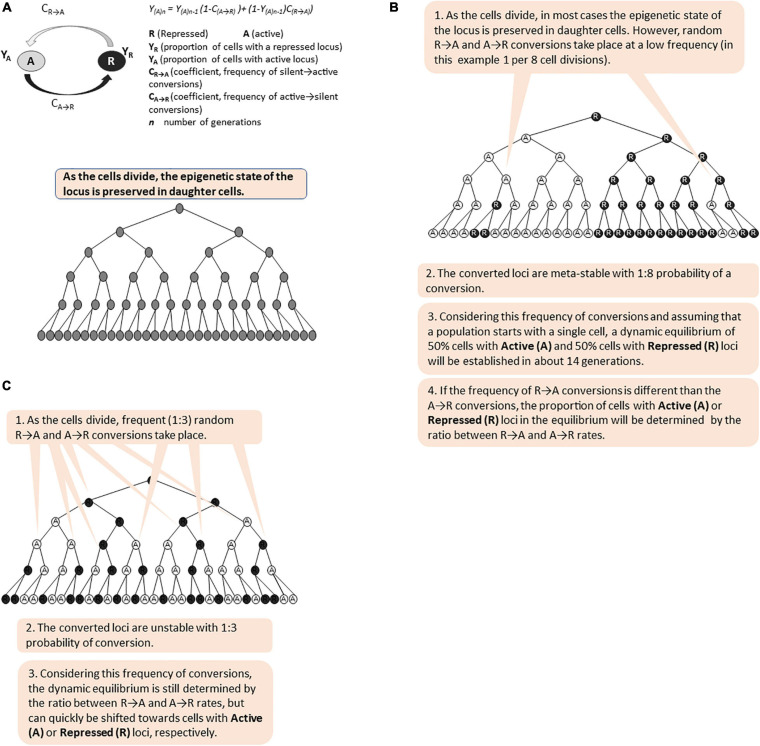
Simulation of long-term and short-term epigenetic memory. The figure is available online as an animated PowerPoint^®^ file to better present the sequence of events in the described processes. **(A)** Calculation of the proportion of cells (Y) with active (A) and repressed (R) state of a given gene in a population of cells. **(B)** A meta-stable locus with low frequency of epigenetic conversions. **(C)** Unstable bi-modal locus with high frequency of epigenetic conversions.

Short- and long- term epigenetic memories differ in the frequency of the conversions between the active (A) and repressed (R) states (Figure 5). We and others have previously introduced algorithms to calculate the proportion of cells with an active or repressed expression state at a given locus (Jeffery et al., 2013; Mano et al., 2013). Both algorithms postulate that the switches from Active to Repressed state (A→R) are independent of the frequency of switches from Repressed to Active state (R→A) regardless of the factors contributing to these switches, and that there is less than one switch per generation. Given this, and assuming that a population of cells originates with a single cell (or all cells in the original population are at only Active or Repressed state, respectively), the algorithms can calculate the proportions of cells with a given Active or Silent locus after certain number of cell divisions. In Figure 5, we present simulations of short- and long-term memory based on these algorithms. These simulations demonstrate that the proportions of cells with active (A) and repressed (R) states are determined by the ratio of A→R versus R→A frequencies and not by the frequencies themselves. Importantly, both short- and long- term memory will ultimately produce phenotypic variation and should both be considered as bet-hedging strategies in the expectation of a change. It remains to be established under what circumstances one or the other strategy is more beneficial to the cell population. Further, there is no solid evidence or opinion if these strategies complement each other and if there is a molecular mechanism that coordinates them. We have already expressed the opinion that shared repressive or activating co-factors such as Rpd3p, Hat1p, Tup1p-Cyc8p, and SWI/SNF could fulfill this function. Mathematical modeling approaches similar to the ones used to analyze the bimodality of *GAL* gene expression could be employed to address these very interesting questions.

### Aging and Bet-Hedging

Replicative aging in *S. cerevisiae* and other species is associated with reduced expression of histones, loss of heterochromatin and global up-regulation of transcription (Feser et al., 2010; Hu et al., 2014; Song and Johnson, 2018). In the context of the variation and variegation phenomena described above, the reduced stability of chromatin and gene repression means that more pronounced bimodality and/or meta-stability with more frequent “on/off” conversions can take place. Hence, populations of aging cells could be more heterogenic and could consequently have better adaptability to changes in the environment. Indeed, an earlier study has suggested that such scenario is feasible (Levy et al., 2012). The synthesis of trehalose (a branch in the synthesis of glycogen) is a key response to stress, including heat-shock. The authors have found that populations of aging cells display a higher level of phenotypic heterogeneity and a better survival in heat, and correlated these effects to the bimodal expression of *TSL1*, a component of the trehalose-6-P synthase/phosphatase complex (Levy et al., 2012). *TSL1* together with its paralog *TPS3* are considered stress-resistance genes. A subsequent study proposed that an age-related bet-hedging strategy contributes to heat resistance (Hellweger et al., 2014).

Two recent studies focused on the age-related changes in transcriptional noise (Liu et al., 2017; Sarnoski et al., 2018). The authors compared the intra-cellular variations (these were defined as variations in gene expression in a given cell across several generations) in haploid and diploid cells. It was found that in haploid cells the intra-cellular “noisy” expression was reduced during normal aging (Liu et al., 2017) while in diploid cells intra-cellular variability was relatively stable (Sarnoski et al., 2018). However, in both haploid and diploid cells this period was followed by a catastrophe phase of several generations in which noise increased (Liu et al., 2017; Sarnoski et al., 2018). Experimental data and computational simulations suggested that this increase could be related to the aging-associated increase in chromatin state transitions and chromatin instability (Liu et al., 2017; Sarnoski et al., 2018). These studies were limited to the analysis of constitutive promoters, thus leaving open the possibility that inducible promoters may display different noise behavior. Nevertheless, theses studies provide significant details on the dynamics of cell-to-cell variations in aging populations and suggest that epigenetic control is reduced in old cells, consequently boosting their adaptivity. It will be very interesting to use similar approaches and compare noise and growth rates of young and aging populations upon shifts in carbon source, nutrient deprivations and other metabolic stressors.

## Concluding Remarks

Phenotypic heterogeneity amongst the individual cells in a population is a strategy that aids adaptation to the changing environment and maintains “memory” for past exposures. This review has summarized our current understanding of the molecular basis of phenotypic heterogeneity at the epigenetic level in *S. cerevisiae.* Specifically, it focused on the contributions of gene regulatory circuits and epigenetic mechanisms to variations in gene expression. Heterogenic gene expression has also been observed in metazoan, with implications to development and homeostasis. The knowledge acquired in budding yeast can be used as a foundation for better understanding of the significance of cell-to-cell variations in gene expression in all eukaryotes.

## Author Contributions

KS and SMS prepared the figures and wrote a draft of the manuscript. KY wrote the final version of the manuscript. All authors contributed to the article and approved the submitted version.

## Conflict of Interest

The authors declare that the research was conducted in the absence of any commercial or financial relationships that could be construed as a potential conflict of interest.
